# Data Privacy in Medical Informatics and Electronic Health Records: A Bibliometric Analysis

**DOI:** 10.1007/s10728-025-00519-0

**Published:** 2025-05-14

**Authors:** Kemal Hakan Gulkesen, Esra Tokur Sonuvar

**Affiliations:** Fac Med, Dept Biostat & Med Informat, Akdeniz Univ, Antalya, Turkey

**Keywords:** Medical informatics, Data security, Bibliometrics, Text mining, Cluster analysis

## Abstract

This study aims to evaluate scientific publications on “Medical Informatics” and “Data Privacy” using a bibliometric approach to identify research trends, the most studied topics, and the countries and institutions with the highest publication output. The search was carried out utilizing the WoS Clarivate Analytics tool across SCIE journals. Subsequently, text mining, keyword clustering, and data visualization were applied through the use of VOSviewer and Tableau Desktop software. Between 1975 and 2023, a total of 7,165 articles were published on the topic of data privacy. The number of articles has been increasing each year. The text mining and clustering analysis identified eight main clusters in the literature: (1) Mobile Health/Telemedicine/IOT, (2) Security/Encryption/Authentication, (3) Big Data/AI/Data Science, (4) Anonymization/Digital Phenotyping, (5) Genomics/Biobank, (6) Ethics, (7) Legal Issues, (8) Cloud Computing. On a country basis, the United States was identified as the most active country in this field, producing the most publications and receiving the highest number of citations. China, the United Kingdom, Canada, and Australia also emerged as significant countries. Among these clusters, “Mobile Health/Telemedicine/IOT,” “Security/Encryption/Authentication,” and “Cloud Computing” technologies stood out as the most prominent and extensively studied topics in the intersection of medical informatics and data privacy.

When the publications are divided into five periods, ‘Mobile Health/Telemedicine/IOT’ and ‘Cloud Computing’ categories are significantly prominent in the late periods, while ‘Ethics’ topic is less prominent in the late periods.

## Introduction

Scientific and technological developments have increased the importance and prevalence of health information systems. These systems are critical to both improving patient care and supporting health research [15]. However, the increasing use of these systems brings with it a number of new challenges, especially in terms of data privacy and security. For clarity, this study distinguishes between ‘data privacy,’ which concerns the control over access to personal information; ‘data security,’ which focuses on protecting data from unauthorized access or breaches [2]; and ‘information security,’ encompassing broader measures to ensure the confidentiality, integrity, and availability of data. Within this framework, this study aims to examine research on data privacy on medical informatics and electronic health records.

The use of electronic health records (EHR) is increasing significantly. Naturally, digital health information databases contain sensitive patient information. Despite the expected benefits from EHRs, there are security concerns regarding the confidentiality and privacy of patients’ personal information [12, 21, 16]. Studies have shown that robust privacy regulations, such as the Health Insurance Portability and Accountability Act (HIPAA) and the General Data Protection Regulation (GDPR), play a crucial role in mitigating these risks [4, 23].

Studies in Medical Informatics are important for understanding global trends and examining historical developments in depth [10]. A study by Gülkesen and Haux (2019) aimed to identify the main research topics and trends in medical informatics research based on core medical informatics journals. In this study, it was observed that there were new developments in the context of mobile healthcare and clinical informatics as well as classical topics in medical informatics [7].

In a systematic review, it is aimed to summarize the studies carried out in the field of Health Information Management (HIM) and Health Informatics and to present the best articles published in this field in 2020. According to this review, data-related issues in electronic health records are a major part of the HIM research literature [3].

A year later, a scoping review by Hussain et al. (2021) examined qualitative empirical research published in JAMIA (Journal of the American Medical Informatics Association) from 1994 to 2019. This review indicates that, despite a rich tradition of qualitative research in JAMIA, the application of these methods has remained limited [11]. In light of these studies, a systematic analysis of approximately 1,000 publications was conducted to determine the patient-centered care trend supported by AI and machine learning in the Clinical Information Systems section of the IMIA Medical Informatics Yearbook. The findings indicate that the term “Covid” has recently gained significant importance in the field of telemedicine [8].

A bibliometric analysis of 34,742 articles obtained from WoS (2011–2020) mapped the developments and research trends in medical informatics over the last decade. According to this analysis, the most productive author was found to be David W Bates, the most productive institution was Harvard University, and the most productive country was the USA [9].

Siamian and colleagues (2023) investigated the scientific output and collaboration networks in the disciplines of medical informatics, health information management, medical librarianship, and information sciences in Iran [22].

This literature analysis was conducted to examine the trends and subjects of privacy and security studies of medical informatics. With developing technologies and new research methods, studies are expected to evolve and change continuously [6]. Unlike previous studies that focused on a limited time frame or thematic scope, this study spans a broader temporal range (1975–2023) and employs novel clustering methods to analyze evolving themes, including ethical and legal issues.

This study covers 7,165 articles in the fields of medical informatics and privacy between 1975 and 2023 and reveals a significant increase in the publication trends of these articles over time, especially from 2018 onwards. With this study, it is aimed to understand the past status of data privacy studies in the field of medical informatics and therefore the future direction.

## Methods

### Data

A search was conducted in the Web of Science (WoS) database on June 15, 2023, for articles indexed in the Science Citation Index Expanded (SCIE) using the query ALL=((“health informatics” OR “medical informatics” OR “health records” OR “Clinical Informatics” OR “healthcare data” OR “health data” OR “medical data” OR “biomedical data” OR genetic OR genomic OR genome OR “health information systems” OR “hospital information systems” OR “Medical Records” OR “Medical Order Entry Systems” OR “Health Information Exchange” OR HIPAA OR mhealth OR telemedicine OR telehealth OR “health technologies” OR “wearable devices” OR “health monitoring” OR “Health Smart Cards”) AND privacy).

In our study, literature on medical informatics and data privacy were analyzed and images were created by using the 1.6.19 version of VOSviewer software for bibliometric analysis (https://www.vosviewer.com/). With the help of this software, text mining was also done in the title and abstracts of the articles.

### Text Mining

With Text mining, we aimed to obtain terms related to the contents of the articles. This functionality of the VOSviewer platform is based on the Apache OpenNLP infrastructure. With this infrastructure, terms that could be one to four words were detected. These terms were grouped together with binary technique and co-word analysis. In this process, VOSviewer software was used for conceptual analysis, co-word grouping, and visualization of results. In the first step, terms that had used more than 20 times were determined and semantically synonyms were combined. The VOSviewer Thesaurus feature was used to combine synonyms. This process enabled a more structured and effective analysis of the literature. After this stage, the 100 most used terms were selected.

As a result of text mining, top 100 terms were examined and 57 of them were obtained by filtering 43 non-specific terms (privacy, data privacy, COVID 19, healthcare, record etc.). The remaining terms were grouped into eight clusters, with the following distribution: Mobile Health/Telemedicine/IOT (1510 terms), Security/Encryption/Authentication (1729 terms), Big Data/AI/Data Science (817 terms), Anonymization/Digital Phenotyping (407 terms), Genomics/Biobank (362 terms), Ethics (494 terms), Legal Issues (217 terms), and Cloud Computing (251 terms).This process was carried out by the two authors separately and a consensus decision was made by discussing different points of view. Then the articles were grouped into 1975–2003, 2004–2008, 2009–2013, 2014–2018, 2019–2023, the frequencies of the previously detected 57 terms in these groups were examined (Table 1). The time periods were chosen based on significant technological and regulatory milestones. For instance, the widespread adoption of Electronic Health Records (EHRs) in the early 2000s and the introduction of the Health Insurance Portability and Accountability Act (HIPAA) in 1996, which established national standards for protecting patient health information, were pivotal moments influencing data privacy research [5, 19]. 

**Table 1:** Distribution of published articles in the fields of medical informatics and data privacy

	Ethic		Security /Encription /Autentication		Big Data /AI/ Data Science		Cloud Computing		Anonymization /Digital phenotyping		Mobile Health /Telemedicine /IOT		Legal Issues		Genomics /Biobank		Total	
	n	%	n	%	n	%	n	%	n	%	n	%	n	%	n	%	n	%
1975–2003	75	54.3%	25	18.1%	0	0.0%	0	0.0%	3	2.2%	26	18.8%	9	6.5%	0	0.0%	138	100.0%
2004–2008	35	28.0%	36	28.8%	0	0.0%	0	0.0%	9	7.2%	39	31.2%	6	4.8%	0	0.0%	125	100.0%
2009–2013	64	20.6%	84	27.1%	6	1.9%	9	2.9%	35	11.3%	92	29.7%	12	3.9%	8	2.6%	310	100.0%
2014–2018	112	12.4%	232	25.8%	60	6.7%	98	10.9%	69	7.7%	247	27.4%	16	1.8%	66	7.3%	900	100.0%
2019–2023	183	6.5%	735	26.2%	130	4.6%	399	14.2%	127	4.5%	981	34.9%	26	0.9%	229	8.1%	2810	100.0%
Total	469		1112		196		506		243		1385		69		303		4283	

Data visualization and clustering analyses were performed using VOSviewer (version 1.6.19) and Tableau Desktop (version 1.0). Network analysis, such as co-authorship network, co-keywords and co-authorship countries analyses, were also handled with VOSviewer. In visual presentation, the relational intensity method is adopted as a normalization step. The visualization resolution was set as 1.00.

## Results

**Fig. 1:**
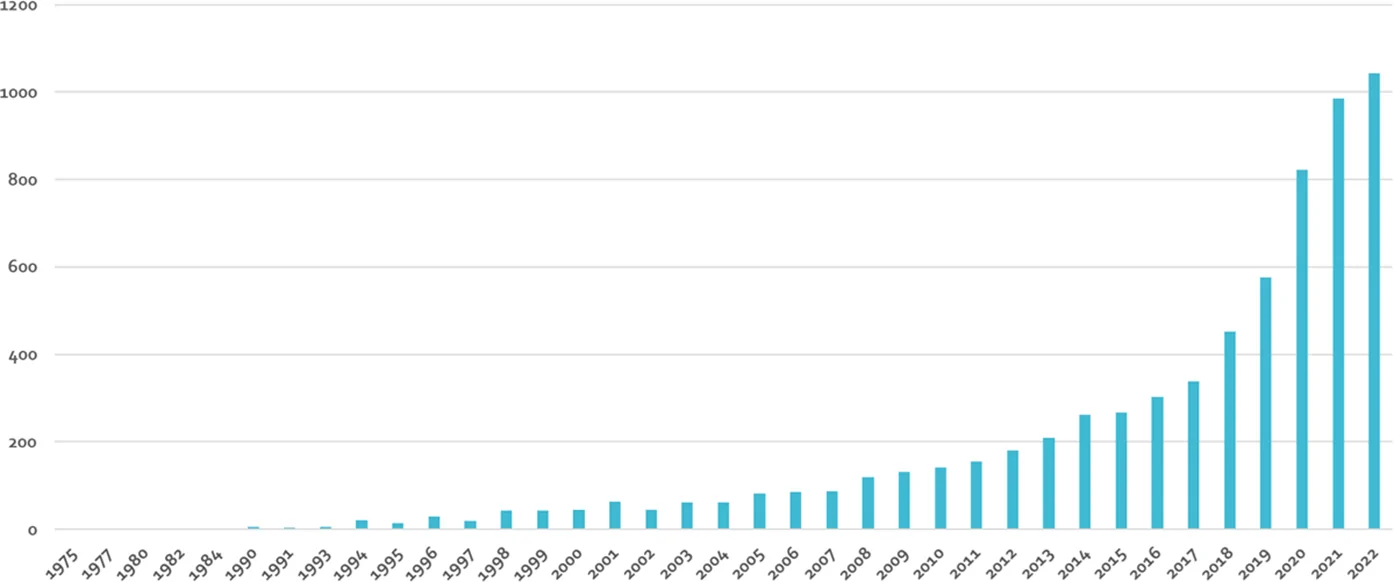
Number of articles written on medical informatics and data privacy from 1975 to 2022

The total number of articles related to the relevant terms is 7,165. Of these, 6.003 (79.47%) papers are original research papers (Table 2). In the first years (1975–1977), there were four articles, while in 2022 the number of articles reached 1044. The publication trend has increased significantly since 2018 (Fig. 1).

**Table 2:** Distribution of document type

Document Type *SCIE	*n*	%
Article	6,003	79.47%
Review Article	745	9.86%
Editorial Material	288	3.81%
Proceeding Paper	230	3.04%
Early Access	138	1.83%
Letter	42	0.56%
News Item	42	0.56%
Meeting Abstract	27	0.36%
Book Chapters	20	0.26%
Correction, Book Review, Data Paper, Correction, Addition, Note, Reprint, Retracted Publication, Retraction, Software Review	19	0.25%
Total	7165	100.00%

7,165 articles written between 1975 and 2023 have been cited 162,715 times. The average citation is 22,7, per article.

**Fig. 2:**
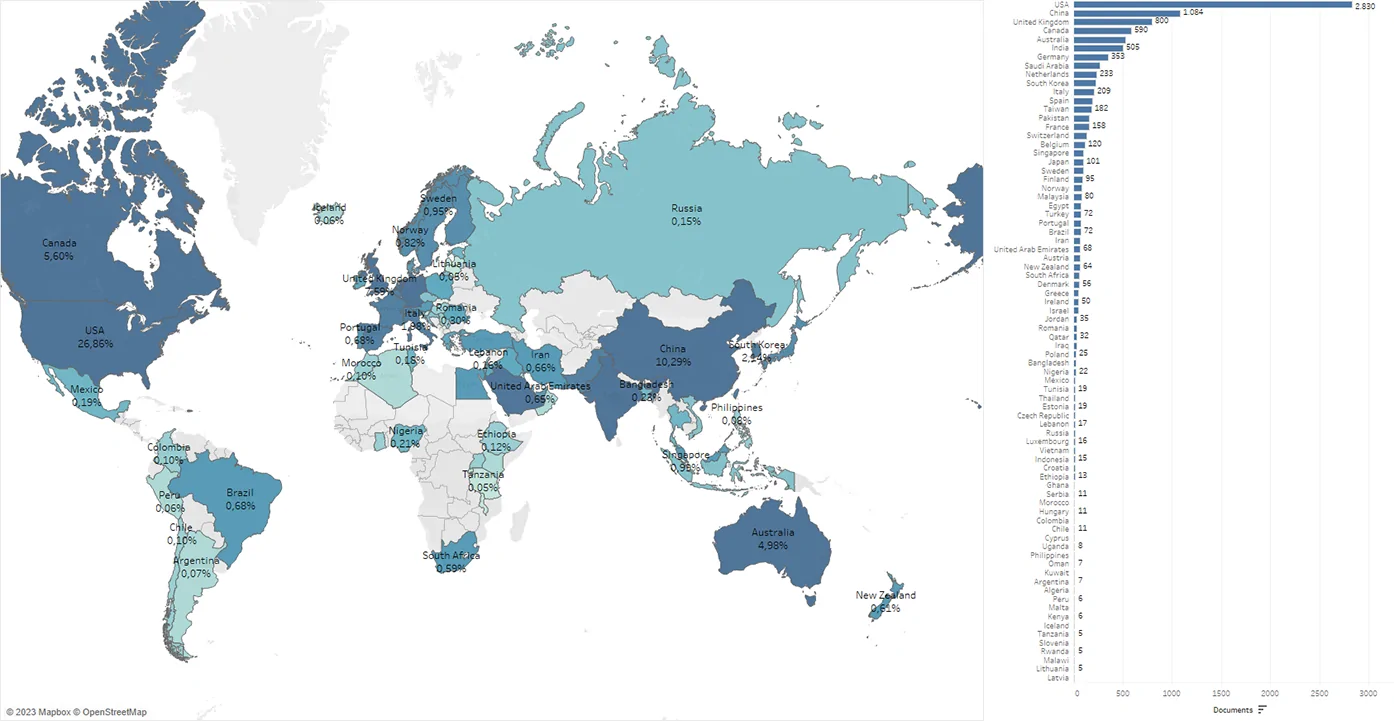
The share of countries in the total number of publications in the field

From 117 countries, the top five countries with the highest number of publications are as follows: The United States leads with 2830 (33.4%) publications, followed by China with 1084 (10.3%) publications, the United Kingdom with 800 (7.8%) publications, Canada with 590 (5.6%) publications and Australia with 505 (5.0%) publications. This distribution and the number of publications of other countries are shown in detail in Fig. 2.

**Fig. 3:**
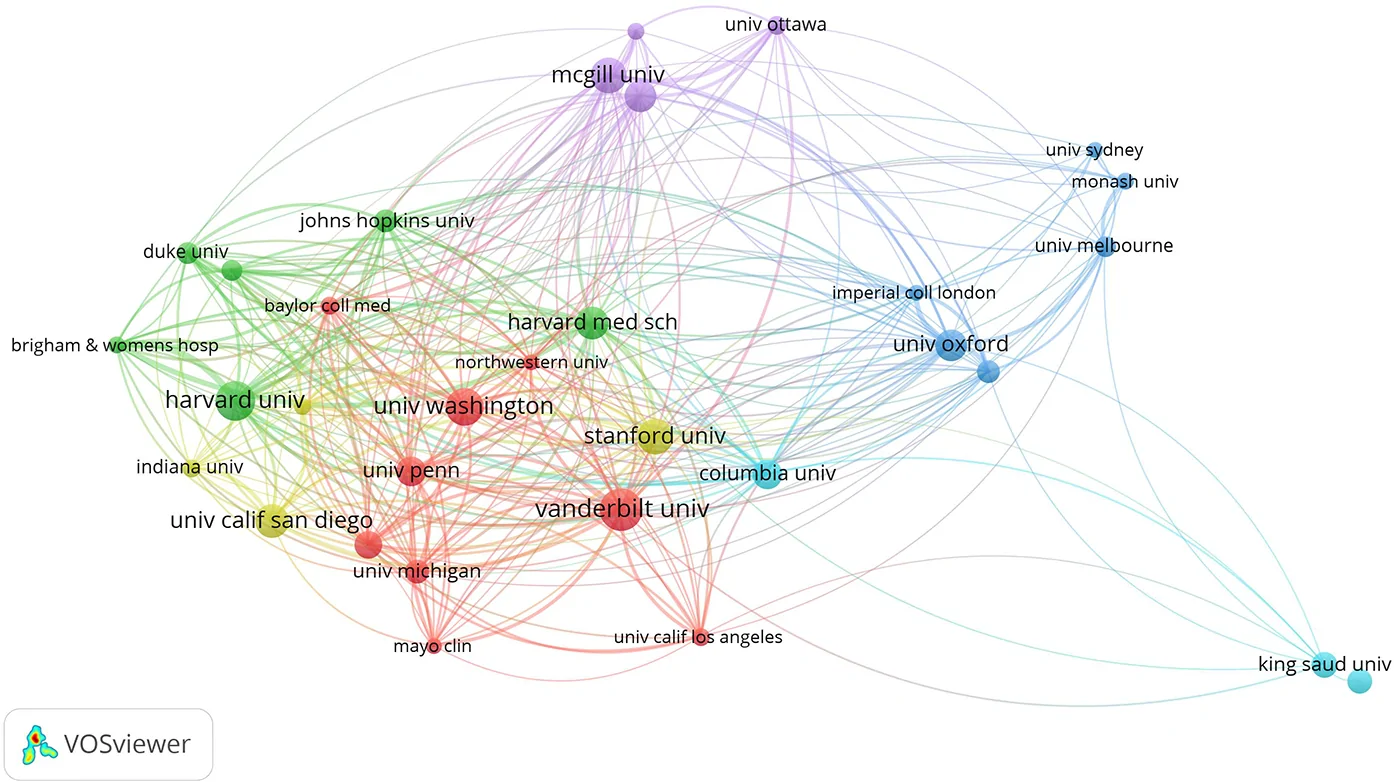
Network among organizations based on co-authorship document counts

In this analysis, citations data from 7367 different organizations were examined. The analysis focused on organizations with at least 20 citations and 20 documents and used a threshold of 149. Harvard University stands out as the institution with the highest number of documents, indicating extensive research activities and great scientific productivity in this field. Likewise, Vanderbilt University has the highest number of citations and total link strength, which indicates that this organization has a significant scientific impact. Other leading institutions include Washington University, University of California San Diego and McGill University. These organizations are influential and respected in their fields, with high document and citation counts (Fig. 3).

While the most terms are available in Cluster 2(Security /Encription/ Autentication) and cluster 1 (Mobile Health /Telemedicine /IOT), the least term appears to be in cluster 7 (Legal Issues). The terms in the eight clusters detected are shown in Table 3.

**Table 3:** Cluster analysis of terms in medical informatics and data privacy

Cluster 1		Cluster 2		Cluster 3		Cluster 4		Cluster 5		Cluster 6		Cluster 7		Cluster 8	
Mobile Health /Telemedicine /IOT	*n*	Security /Encription/ Autentication	*n*	Big Data/ AI/ Data Science	*n*	Anonymization /Digital phenotyping	*n*	Genomics /Biobank	*n*	Ethic	*n*	Legal Issues	*n*	Cloud Computing	*n*
telemedicine	340	security	445	machine learning	173	privacy-preserving	81	genomics	95	ethics	236	hipaa	146	cloud computing	203
mhealth	259	blockchains	378	deep learning	131	privacy preservation	76	genetic testing	91	informed consent	140	regulations	38	edge computing	48
internet of things	275	confidentiality	204	artificial intelligence	128	differential privacy	60	genetics	64	consent	62	gdpr	33		
telehealth	157	authentication	144	big data	121	anonymity	53	bioinformatics	40	research ethics	56				
mobile health	108	encryption	134	federated learning	115	anonymization	53	biobanks	36						
mobile applications	106	access control	124	genetic algorithms	74	de-identification	43	genetic privacy	36						
mobile phones	82	cryptography	85	data mining	39	privacy preserving	41								
smartphone	66	homomorphic encryption	76	computational modeling	36										
internet of medical things	64	data protection	73												
sensors	53	data security	66												
**Total**	**1510**		**1729**		**817**		**407**		**362**		**494**		**217**		**251**

**Fig. 4:**
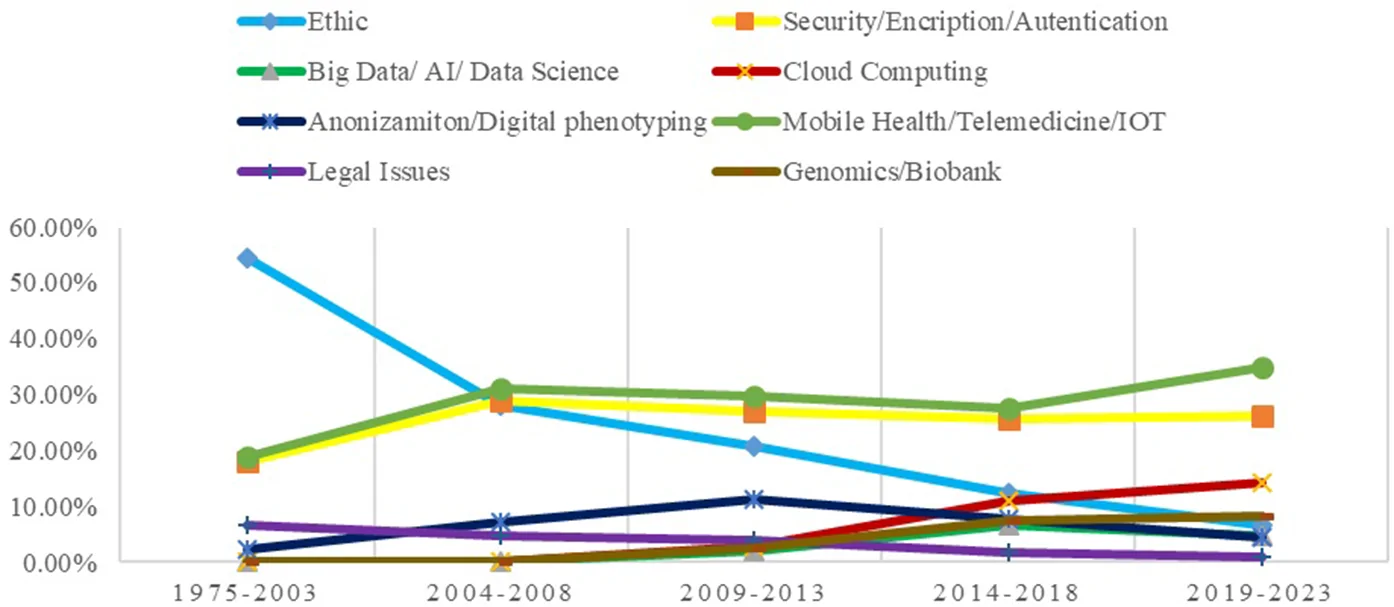
Evolution of research areas in medical informatics and data privacy from 1975 to 2023

Figure 3 illustrates how the distribution of clusters in the literature has evolved over time. The change in research trends in medical informatics and privacy between 1975 and 2023 can be clearly seen in (Fig. 4). The chart shows that the Mobile Health/Telemedicine/IOT (18.84%) and Ethic (54.35%) clusters took up significant space between 1975 and 2003, but the Security/Encryption/Authentication (18.12%) and Cloud Computing (0.00%) clusters found relatively little space. In 2004–2008, the share of the categories Mobile Health/Telemedicine/IOT (31.20%) and Ethics (28.00%) decreased compared to the previous period, while the share of the Security/Encryption/Authentication (28.80%) increased. In 2009–2013, the Big Data/AI/Data Science (1.94%) and the Genomics/Biobank (2.58%) also appeared on the chart, and the Legal Issues increased significantly with 3.87%. In 2014–2018, the Big Data/AI/Data Science was 6.67%. Finally, in 2019–2023, the Cloud Computing (14.20%) and the Genomics/Biobank (8.15%) clusters took up significant space and showed a huge increase. As a result, these figures show how much research and technological developments in these fields have accelerated.

## Discussion

In this study, it has been observed that the number of articles on data privacy has increased every year and changes have occurred in the subjects of the articles.

In the first three years (1975–1977), there were only four articles on data privacy, while in 2022, 1044 articles were published. These data show that in the 70s, data privacy was a marginal issue that only a small number of researchers were interested in. Over time, with the widespread use of electronic health records, privacy has increasingly become a tangible concern in daily life due to the challenges in securing sensitive patient data, rather than remaining a theoretical and philosophical issue. The enactment of regulatory frameworks such as HIPAA (1996) and GDPR (2018) has significantly influenced the trajectory of research on data privacy in medical informatics [23]. These regulations have established stringent guidelines on data protection, requiring healthcare providers to implement security measures and encryption protocols to safeguard patient information [21]. Therefore, research on the subject has seen a corresponding increase. The publication trend shows a significant increase in the number of articles on data privacy after 2018. Looking at the trend in Fig. 1, it is seen that this increase continues. Therefore, it can be said that data privacy is an issue of increasing importance every year.

The 2022 study, “Global Scientific Research Landscape on Medical Informatics From 2011 to 2020: Bibliometric Analysis,” found that the United States was the most productive country in medical informatics between 2011 and 2020, followed by Germany, the United Kingdom, China, and Canada [9]. In the field of medical informatics and data privacy, the top five countries with the highest number of publications are the United States of America, China, the United Kingdom, Canada, and Australia, respectively. It is noteworthy that Germany is not on this list. The reason for this may be that the digitalization of health data in Germany is slower than in many developed countries [1].

When the productivity of the institutions is analyzed, Harvard University, Vanderbilt University, Washington University, University of California San Diego from the USA ranked in the first four places, while the University of McGill from Canada ranked fifth. These institutions have an influential and respected position in their fields with high document and citation numbers. When we look at the geographical distribution of publications, the leadership of the United States of America in this field confirms that this country has assumed a pioneering role in this domain. However, the contributions of countries such as China, the UK, Canada and Australia are also noteworthy. When examining geographic differences, the United States emerges as a leader in ‘Mobile Health/Telemedicine/IOT,’ driven by regulatory frameworks such as HIPAA and extensive federal funding for digital health technologies. In contrast, China’s leadership in ‘Cloud Computing’ research can be linked to state-led initiatives promoting data-intensive technologies [9, 17]. This geographical distribution shows that these countries can promote potential collaborations and joint studies on medical informatics and data privacy. In Europe, the emphasis on ‘Data Privacy and Security’ research can be attributed to the implementation of robust regulatory frameworks like the General Data Protection Regulation (GDPR). Similarly, India’s focus on ‘Telemedicine’ reflects the country’s push for digital health initiatives like the National Digital Health Mission (NDHM), aimed at improving healthcare access across its vast population. The United Kingdom, on the other hand, has emerged as a leader in ‘AI and Data-Driven Healthcare,’ driven by NHS open data initiatives and substantial government investment in AI technologies [15, 23].

Data privacy in medical informatics has become increasingly important in recent years, and this study is a comprehensive reflection of the scientific contributions of this trend in this field. The prominence of data privacy related terms in the research results, especially in clusters such as “Security/Encryption/Authentication” and “Anonymisation/Digital Phenotyping”, clearly shows the place of this topic in medical informatics [13]. The identified clusters reflect the interplay between technological advancements, policy initiatives, and global health priorities. These variations underscore how local priorities, resources, and regulations shape global research directions. For example, while high-income countries focus on advancing data-driven technologies, emerging economies like India prioritize telemedicine to address healthcare accessibility challenges. Similarly, Europe’s strong emphasis on privacy and security reflects its regulatory framework, particularly GDPR, which has been pivotal in guiding ethical and legal discussions in the field [23]. For instance, the prominence of the ‘Mobile Health/Telemedicine/IOT’ cluster can be attributed to the widespread adoption of smartphones and mobile health applications, particularly in high-income countries like the United States, where significant investment in digital health technologies has been made [9]. Similarly, the increasing focus on ‘Cloud Computing’ aligns with the global shift toward cloud-based solutions for secure data storage and processing, driven by both technological advancements and the demand for scalable healthcare data infrastructure [18].

The observation of a significant increase in publications in all clusters, especially in the 2014–2018 and 2019–2023 periods, indicates that research in this field has gained momentum both with the acceleration of technological developments and the impact of the global epidemic process. Advancements in cloud computing, AI-driven healthcare applications, and blockchain-based security models have played a pivotal role in reshaping the landscape of medical informatics research [11]. Furthermore, the COVID-19 pandemic has accelerated digital transformation efforts in healthcare, increasing the demand for remote patient monitoring and telemedicine solutions, which, in turn, has raised concerns about data privacy and security [14]. This increase also shows that medical informatics is becoming increasingly complex and multidimensional, especially in terms of data privacy. While ethical and legal issues were covered more than other topics in the beginning years, their proportion in total studies has decreased over the years.

The ethical and legal clusters, while prominent in the early years, have shown a diminishing trend over time. This decline may be attributed to a perceived resolution of foundational issues in the initial stages of research, as well as a shift in focus toward addressing practical and technical challenges such as security and encryption. However, recent studies indicate that ethical concerns in AI-driven healthcare and big data analytics remain critical, particularly in areas such as algorithmic bias, patient consent, and data ownership [15]. As AI and predictive analytics become more integrated into healthcare systems, the need for updated ethical frameworks addressing fairness, transparency, and accountability is becoming increasingly evident [20]. Moreover, the rapid development of AI-driven methods and cloud computing has redirected research priorities, leaving less emphasis on ethical and legal aspects. Despite this decline, ethical considerations remain critical, particularly as advanced technologies raise new concerns regarding privacy, fairness, and patient autonomy. Addressing these issues will continue to be essential for the responsible implementation of technical solutions in healthcare. This shift may be attributed to the growing emphasis on solving practical challenges posed by technological advancements, such as security and encryption, rather than discussing foundational ethical frameworks. However, ethical concerns remain a critical aspect of medical informatics, particularly as data-driven approaches like AI continue to raise questions about privacy, fairness, and patient autonomy [15]. We believe that the reason for this is that the practical problems arising with the increasing use of electronic health records in health service delivery and the impact of the development of technology and other issues occupy more space on the agenda. For example, with the widespread use of smart phones, studies in the field of mobile health have become mandatory.

## Limitations

Our study has some limitations. Firstly, only articles indexed in the SCIE database were analyzed, and data were exclusively obtained from the Web of Science (WoS). Consequently, other widely used databases such as Scopus, PubMed, and Google Scholar were not included, which may have led to the exclusion of relevant publications. Furthermore, proceedings from key conferences were also excluded from the scope of this study, which might limit the comprehensiveness of the analysis.

In the part of the study regarding text mining, subjective interpretations of the authors may affect the results. The authors eliminated terms that they did not consider directly related to a topic, and also categorised the terms into clusters according to their own domain knowledge and experience. Although the results obtained with other researchers may not show dramatic changes, some different results may be obtained.

This study shows the situation in mid-2023. Due to the rapid developments in the field of informatics, the picture may change in a few years. Therefore, it is important to follow the developments in this field in the future and to identify new trends. We are in a period of rapid development of machine learning and text mining. With the help of developing technologies, new studies that bring different perspectives to the field can be carried out in the near future.

## Conclusion

Data privacy in medical informatics has become an increasingly important area in parallel with technological developments. Both the acceleration of technological developments and the electronic storage and sharing of health information make research in this field compulsory. In recent years, issues such as cloud computing, blockchain technologies, machine learning and social media privacy have been considered in the context of data privacy in medical informatics. While it is observed that the United States of America stands out in this field, we see that data privacy in medical informatics is a global issue and many countries are actively working on this issue. In the future, with technological advances, we anticipate that more comprehensive research will be conducted on data privacy in medical informatics and new concepts and approaches will emerge in this field.
